# RNA Viral Metagenome Analysis of Subnanogram dsRNA Using Fragmented and Primer Ligated dsRNA Sequencing (FLDS)

**DOI:** 10.1264/jsme2.ME20152

**Published:** 2021-05-01

**Authors:** Miho Hirai, Yoshihiro Takaki, Fumie Kondo, Masayuki Horie, Syun-ichi Urayama, Takuro Nunoura

**Affiliations:** 1 Super-cutting-edge Grand and Advanced Research (SUGAR) Program, Japan Agency for Marine Science and Technology (JAMSTEC), 2–15 Natsushima-cho, Yokosuka, Kanagawa 237–0061, Japan; 2 Hakubi Center for Advanced Research, Kyoto University, 53 Kawahara-cho, Shogoin, Sakyo, Kyoto 606–8507, Japan; 3 Institute for Frontier Life and Medical Sciences, Kyoto University, 53 Kawahara-cho, Shogoin, Sakyo, Kyoto 606–8507, Japan; 4 Laboratory of Fungal Interaction and Molecular Biology, Department of Life and Environmental Sciences, University of Tsukuba, 1–1–1 Ten-no-dai, Tsukuba, Ibaraki 305–8577, Japan; 5 Microbiology Research Center for Sustainability, University of Tsukuba, 1–1–1 Ten-no-dai, Tsukuba, Ibaraki 305–8577, Japan; 6 Research Center for Bioscience and Nanoscience (CeBN), JAMSTEC, 2–15 Natsushima-cho, Yokosuka, Kanagawa 237–0061, Japan

**Keywords:** metagenomics, subnanogram dsRNA, virome

## Abstract

Fragmented and primer ligated dsRNA sequencing (FLDS) is a sequencing method applicable to long double-stranded RNA (dsRNA) that enables the complete genome sequencing of both double- and single-stranded RNA viruses. However, the application of this method on a low amount of dsRNA has been hindered by adaptor dimer formation during cDNA amplification and sequence library preparation. We herein developed FLDS ver. 3 by optimizing the terminal modification of an oligonucleotide adaptor and the conditions of adaptor ligation. We also examined the concentration of Mg^2+^ in the PCR reaction for cDNA amplification and the purification method of amplified cDNA. Fine sequence reads were successfully obtained from metagenomic shotgun sequencing libraries constructed from 10 and 100 pg dsRNA, and these libraries exhibited weaker detection sensitivity for low-abundance dsRNAs (viral genomes and genome segments) than that constructed from 1‍ ‍ng of dsRNA. We also report the utility of capillary electrophoresis for dsRNA quantification. The FLDS ver. 3 package expands the frontiers of our knowledge in RNA virus diversity and evolution.

Long cellular dsRNA is a genome of double-stranded RNA (dsRNA) viruses and a replicative intermediate of single-stranded RNA (ssRNA) viruses (Morris and Dodds, 1979). Therefore, with the exception of retroviruses, long cellular dsRNA is recognized as a hallmark of the presence of RNA viruses, and the RNA sequencing of long cellular dsRNA is an efficient approach for assessing RNA viromes associated with certain organisms (Roossinck *et al.*, 2010; Decker *et al.*, 2019). To the best of our knowledge, the combination of dsRNA purification and “fragmented and primer ligated dsRNA sequencing” (FLDS) is the most efficient method for evaluating RNA viral genomes, and enables the enrichment of RNA viral sequences and retrieval of complete genome sequences that include both terminal ends (Urayama *et al.*, 2016, 2018). These features of FLDS are advantageous for the *in silico* reconstruction of segmented viral genomes because each segment shares terminal sequences and also for the subsequent identification of viral genes that do not show significant similarity with the genes in public databases (Urayama *et al.*, 2018, 2020). Moreover, this method is capable of efficiently detecting the genetic diversity of isolated virus strains (Kadoya *et al.*, 2020).

FLDS consists of the following four steps: the mechanical fragmentation of dsRNA by ultrasonication, the ligation of a single-stranded oligonucleotide adaptor (cDNA synthesis adaptor) to the 3′ termini of fragmented dsRNA, reverse transcription from adaptor sequences, and cDNA amplification. In FLDS ver. 1, a looped oligonucleotide adaptor harboring phosphorylated and hydroxylated 5′ and 3′ ends, respectively, was used for the reverse transcription of dsRNA (Urayama *et al.*, 2016). To increase the efficiency of cDNA synthesis, an oligonucleotide adaptor with phosphorylated ends was ligated to the 3′ end of dsRNA prior to cDNA synthesis in FLDS ver. 2 (Urayama *et al.*, 2018). A recent study reported that the 3′-end of mechanically-sheared dsDNA carries a phosphate group, hydroxyl group, and unidentified structures (Ohtsubo *et al.*, 2019). This finding suggests that a reassessment of the terminal residue modifications in oligonucleotide adaptors will increase the efficiency of adaptor ligation and subsequent cDNA synthesis in FLDS if similar structures occur in mechanically-sheared dsRNA.

In the present study, we developed FLDS ver. 3, which enhanced the efficiency of adaptor ligation and subsequent cDNA synthesis, using an oligonucleotide adaptor with optimized terminal modifications. We also examined the impact of the amount of initial dsRNA and PCR amplification conditions during cDNA synthesis. During this process, the capacity of capillary electrophoresis for the fluorescent quantification of dsRNA was also assessed to estimate the amount of dsRNA.

## Materials and Methods

### Adaptors for initial cDNA synthesis

A U2 oligonucleotide (5′-GAC GTA AGA ACG TCG CAC CA-3′) adaptor carrying various chemical modifications in both termini was synthesized in Nihon Gene Research Laboratories and purified by HPLC. This adaptor was ligated at the 3′-terminus of dsRNA and used as a template sequence in cDNA synthesis. U2 oligonucleotide adaptors were named as follows: U2 oligonucleotide with 5′- and 3′-phosphorylated ends (U2-DNA-5P3P), with 5′-phosphorylated and 3′-deoxylated ends (U2-DNA-5P3H), with 5′-phosphorylated and 3′-aminated ends (U2-DNA-5P3am), and with unmodified 5′-hydroxylated and 3′-deoxylated ends (U2-DNA-5OH3H). A U2 RNA oligonucleotide with 5′-phosphorylated and 3′-deoxylated ends (U2-RNA-5P3H) was also synthesized.

### Quantification of dsRNA

DynaMarker@dsRNA (BioDynamics Laboratory) (0.25‍ ‍μg μL^–1^) diluted with dH_2_O and dsRNA solutions (1, 2.5, 5, 10, 25, and 50‍ ‍ng μL^–1^) was prepared to obtain a standard curve in a Fragment Analyzer System (Agilent Technologies). A Fragment Analyzer DNA/NGS Kit DNF-474-0500 (Agilent) was used for fluorescent quantification. Environmental dsRNA concentrations were measured based on the standard curve.

### FLDS

A previously reported diatom colony (Urayama *et al.*, 2016) stored in JAMSTEC was used as the source of dsRNA. Environmental dsRNA was extracted and purified using a cellulose column method (Urayama *et al.*, 2015; 2016), and further processed with DNase I (Takara Bio) and S1 nuclease (Thermo Fisher Scientific). Nuclease digestion was performed in 130‍ ‍μL of nuclease buffer (final concentrations of 60‍ ‍mM CH_3_COONa, 10‍ ‍mM MgCl_2_, 200‍ ‍mM NaCl, and 2‍ ‍mM ZnSO_4_) with 2U of DNase I and 200U of S1 nuclease. The reaction mixture was then transferred into a small glass vial (microTUBE AFA Fiber Pre-Slit Snap-Cap), and dsRNA was mechanically fragmented with a Covaris M220 ultrasonicator. The following fragmentation conditions were used: run time 40‍ ‍s, peak power 50.0 W, duty factor 2.0%, and 200 cycles/burst. Mechanically shared dsRNA was purified with a Zymoclean Gel RNA Recovery Kit (Zymo Research). The concentrations of purified dsRNA were quantified using the Fragment Analyzer System as described above, and dsRNA was diluted with DNase/RNase-Free water (Nippon Gene) to concentrations ranging between 10 pg μL^–1^ to 1‍ ‍ng μL^–1^.

One microliter of each of the prepared dsRNA solutions was mixed with T4 RNA ligase buffer containing the U2 adaptor (final conc. 0.4‍ ‍μM) and T4 RNA ligase (final conc. 0.8‍ ‍U μL^–1^) (Takara Bio) (final volume: 50‍ ‍μL) in a DNA Lo-Bind Tube (Eppendorf), and incubated at 16°C overnight. The product was purified with a MinElute Gel Extraction Kit (Qiagen) and eluted with 10.5‍ ‍μL buffer solution. To the resultant elute, 0.5‍ ‍μL of 25‍ ‍μM U2-comp primer (5′-TGG TGC GAC GTT CTT ACG TC-3′), a complementary sequence of the U2 adaptor, was added. The mixture was denatured at 95°C for 3‍ ‍min and immediately quenched on ice for 2‍ ‍min thereafter. Eleven microliters of the dsRNA solution was mixed with the reaction mixture of the SMARTer RACE 5′/3′ Kit (Takara Bio) (final volume: 20‍ ‍μL), and cDNA was synthesized according to the manufacturer’s protocol. Residual RNA in the reaction mixture was digested with 12U of RNase H (Takara Bio) at 37°C for 10‍ ‍min.

Synthesized cDNA was further amplified by PCR using KOD-Plus-Neo (Toyobo) and a primer set composed of the U2-comp primer and Universal Primer A Mix (UPM) from the SMARTer RACE 5′/3′ Kit. The final concentrations of the primers, dNTP, and KOD-Plus-Neo polymerase were 0.25‍ ‍μM each, 0.2‍ ‍mM, and 0.02‍ ‍U μL^–1^, respectively. The final volume of the reaction mixture was 50‍ ‍μL. The effects of Mg^2+^ concentrations were examined at 1.5 and 2.5‍ ‍mM, respectively. PCR amplification conditions consisted of initial denaturation at 96˚C for 2‍ ‍min and 25 or 35 amplification cycles at 98°C for 10‍ ‍s, 60°C for 15‍ ‍s, and 68°C for 2‍ ‍min. To remove small DNA fragments (<150 bp), the PCR reaction mixture was mixed with a 70% volume of AMPure XP (Beckman Courter) and purified with elution buffer. Purification was repeated again in the optimization of sequence library preparation.

### Sequencing

The cDNAs obtained were fragmented with Covaris M220. Fragmentation conditions for the 400-bp peak size using microTUBE AFA Fiber Pre-Slit Snap-Cap vials were as follows: run time 60‍ ‍s, peak power 75.0 W, duty factor 15.0%, and 200 cycles/burst. Sequencing libraries were then constructed from fragmented cDNAs with KAPA Hyper Prep Kit Illumina platforms. The quality of the sequence libraries was evaluated using an Agilent 2100 Bioanalyzer with a High-Sensitivity DNA chip and the KAPA library quantification kit. Paired-end sequencing was performed using the Illumina MiSeq platform with a 2×300-bp read length.

### Data processing

Raw Illumina reads were sequentially processed using Trimmomatic ver. 0.39 (Bolger *et al.*, 2014) to trim adaptor sequences and low-quality sequences with an average score for the quality value (QV) <20 over a sliding window of four bases, Bowtie 2 ver. 2.3.5.1 (Langmead and Salzberg, 2012) to remove the PhiX sequences used as an internal control, Cutadapt ver. 2.4 (Martin, 2011) to trim cDNA synthesis adaptors, PRINSEQ ver. 0.20.4 (Schmieder and Edwards, 2011) to exclude low-complexity reads and PCR duplicates, and SortMeRNA ver. 2.1b (Kopylova *et al.*, 2012) to remove rRNA-derived reads. These processes were controlled by a custom Perl script (https://github.com/takakiy/FLDS). Clean reads from each library were normalized by downsampling 0.8 M of paired-end reads using SeqTk ver. 1.3-r106 (https://github.com/lh3/seqtk).

To efficiently exclude the reads derived from host organisms and laboratory contaminants, datasets of pooled sequence reads were prepared because the formation of more and longer contigs in assembly was expected. Taxonomy assignments for each read were then performed using a BLASTX search of the NCBI non-redundant (nr) database. We constructed datasets of pooled reads for each stage to improve the FLDS method in consideration of the computation time for assembly and the removal efficiency of contamination reads. Dataset 1 consisted of reads from 9 libraries constructed with the 5 tested adaptors used for cDNA synthesis. Dataset 2 contained reads from 3 libraries constructed with the U2-DNA-5P3H adaptor at 1‍ ‍ng, 100 pg, and 10 pg of input dsRNA. Each dataset was assembled using CLC Genomic Workbench ver 11 (Qiagen) with the following parameter settings: *k*-mer value of 39 bp, bubble size of 500 bp, and map read back to the contigs with a length fraction of 0.9 and similarity fraction of 0.9. The resulting contigs were characterized as viral, prokaryotic, eukaryal, and unknown sequences based on a BLASTX search of the nr database. Sequence reads mapped to prokaryotic and eukaryal contigs were then identified as host organisms or potential laboratory contaminants and removed from subsequent analyses. The abundance of excluded sequence reads in each library was not significant, ranging between 0.22 and 1.14% of all reads.

### Preparation of a reference database: RNA viral genomes and genome segments

Sequence reads retrieved from the datasets were reassembled using the CLC assembler with the above settings. Among the contigs obtained, possible RNA viral genomes and genome segments were selected based on a BLASTX search of the nr database. The terminal ends of these contigs were identified as follows (Fig. S1). Only reads with adaptor sequences were selected from Trimmomatic-treated reads using the Cutadapt program and then mapped against reference sequences for RNA virus genomes using Bowtie 2. Ultimately, the aligned read maps on the reference sequence were used to count the 5′ aligned position of mapped reads. In FLDS, the read frequencies on both termini are generally higher than in the central region of the genome (Urayama *et al.*, 2016) (Fig. S2A). Therefore, both termini were assessed by the Smirnov-Grubbs test (*P*<0.05) to detect outliers among these counts (Fig. S2B). Possible RNA viral genomes and genome segments with terminal sequences at both ends were used as reference RNA viral genomes and genome segments in the present study. This workflow was performed with a custom Perl pipeline script (https://github.com/takakiy/FLDS). Genes for RNA viral proteins were identified using the FGENESV0 program (http://www.softberry.com/berry.phtml) and NCBI ORF Finder (https://www.ncbi.nlm.nih.gov/orffinder/) and subsequently annotated with a BLAST analysis using the nr database as a reference with an E-value threshold of≤1×10^–5^.

### Comparison of sequence libraries

To assess the impact of different U2 adaptors and revised methods on FLDS library construction, we estimated two parameters from mapping data: the depth and breadth of coverage. The ‘depth of coverage’ is the average number of times that a nucleotide base in the references is covered by reads. The ‘breadth of coverage’ refers to the proportion of the aligned bases relative to the length of the reference sequence. Sequence reads from each library were aligned on the references using Bowtie 2. Alignments were converted to the bam format using samtools ver. 1.9 (Li *et al.*, 2009). The coverage program in BEDtools ver 2.27.1 (Quinlan and Hall, 2010) was used to estimate the depth and breadth of coverage in each reference sequence. Graphics were produced using either the R base package or ggplot2 package (Wickham, 2016) for figures throughout this manuscript. Line fitting was calculated using locally weighted scatter plot smoothing (loess method) with the stat_smooth program in the ggplot2 package.

### Accession numbers

The sequences obtained in the present study are available in the GenBank database repository (accession nos. BNHM01000001–BNHM01000036) and Short Read Archive database (accession nos. DDBJ:DRA 010893).

## Results and Discussion

### Quantification of dsRNA

To the best of our knowledge, conventional methods for quantifying low amounts of dsRNA have not been reported to date. Sufficient and accurate standard curves were obtained over a range of 1‍ ‍ng to 50‍ ‍ng μL^–1^ (R^2^=0.974, *n*=5) using a Fragment Analyzer System with DynaMarker@dsRNA as a standard solution (Fig. S3). In library preparation, the standard curve was then used to measure the amount of environmental cellular dsRNA isolated from a diatom colony.

### Strand-specific sequencing of FLDS and preparation of reference RNA viral genomes and genome segments

FLDS is a method that is capable of strand-specific RNA sequencing. In the present study, we extracted reads providing signatures of strand direction and used them to define the completeness of RNA viral genomes and genome segments. DNA fragments in the cDNA library constructed by this method were flanked by two distinct adaptor sequences (UPM and U2) oriented to the 5′ and 3′ ends of the original strand, respectively (Fig. S1). Accordingly, we identified the direction of the original sequences by extracting paired-end reads, including U2 sequences, and their subsequent mapping on assembled contigs. This information enabled us to establish whether an assembled contig harbored both termini of the genome/genome segment of the RNA virus. Using the novel informatic pipeline constructed in the present study, 67 contigs among all assemblies were defined as potential complete genomes and genome segments, in which 31 sequences reported by Urayama *et al.* (2016) were included, and used as reference sequences in subsequent analyses (Table S1). Taxonomic groups were inferred based on the taxonomic classification of the top hit RNA viruses in the BLASTX search using the nr database.

### Impact of chemical modifications in oligonucleotides on sequence reads

**Fig. 1.**
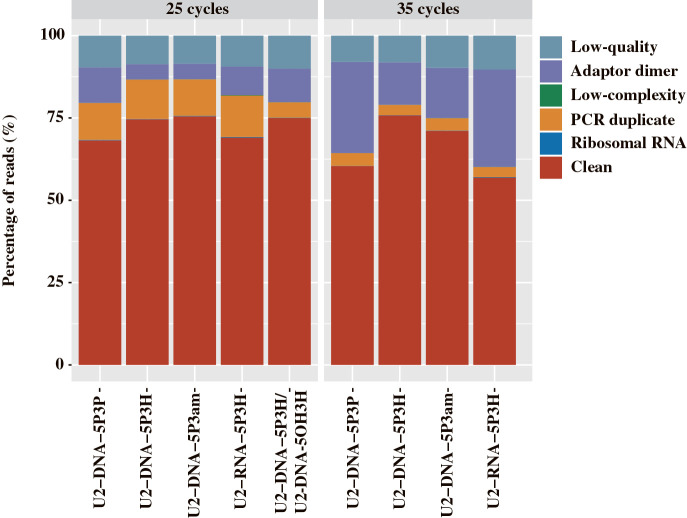
Proportion of reads in sequence libraries constructed with 5 adaptor types. cDNAs synthesized with each oligonucleotide adaptor, except for a mixture of U2-DNA-5OH3H and U2-DNA-5P3H, were constructed with 25 or 35 cycles of cDNA amplification.

To construct high-quality sequencing libraries using low amounts of template dsRNA in FLDS, it is essential to increase the efficiency of adaptor ligation for cDNA synthesis and suppress self-ligation among adaptors. Therefore, we‍ ‍compared five types of oligonucleotide adaptors (U2-DNA-5P3P, U2-DNA-5P3H, U2-DNA-5P3am, U2-DNA-5OH3H, and U2-RNA-5P3H) for cDNA synthesis in the FLDS library construction. Based on the chemical structures of the 3′ end of mechanically-sheared dsDNA (Ohtsubo *et al.*, 2019), oligonucleotides with phosphorylated or hydroxylated 5′-ends were prepared. To prevent ligation among the oligonucleotide adaptors, appropriate chemical modifications were prepared for the 3′-ends. In the initial test, 1‍ ‍ng of mechanically-sheared dsRNA was used as a template, and 25 and 35 PCR cycles were used for cDNA amplification. Unexpectedly, insufficient cDNA amplification was obtained using U2-DNA-5OH3H despite the expected dominance of a phosphate group in the 3′ end of mechanically-sheared dsRNA, as in the case of dsDNA (Ohtsubo *et al.*, 2019). In the sequencing libraries constructed using other U2 adaptors, the abundance of high-quality reads (depicted as Clean in Fig. 1) obtained with 25 and 35 PCR cycles ranged from 60.1–73.8% and 55.0–71.9%, respectively (Fig. 1). The libraries constructed with U2-DNA-5P3H, U2-DNA-5P3am, and a mixture of U2-DNA-5OH3H and U2-DNA-5P3H exhibited better quality than those with U2-DNA-5P3P and U2-RNA-5P3H. In addition, the abundance of the self-ligated products of the oligonucleotide adaptor (depicted as Adaptor dimer in Fig. 1) was higher in libraries constructed from cDNA with 35 cycles of PCR amplification than in those with 25 cycles. Therefore, we used the data sets obtained from cDNAs with 25 cycles of PCR amplification for further analyses.

The assemblies using reads subsampled from each library are summarized in Table 1. Among the libraries, the U2-DNA-5P3H adaptor library produced the greatest number of contigs, total size, average contig length, and N50 value. The rank abundance curves of contigs in each assembly showed that the sequence libraries constructed with U2-DNA-5P3H and U2-DNA-5P3am harbored more contigs with a lower depth of coverage (Fig. S4). In contrast, the library constructed with U2-RNA-5P3H was disadvantaged by the retrieval of contigs with a lower depth of coverage.

**Fig. 2.**
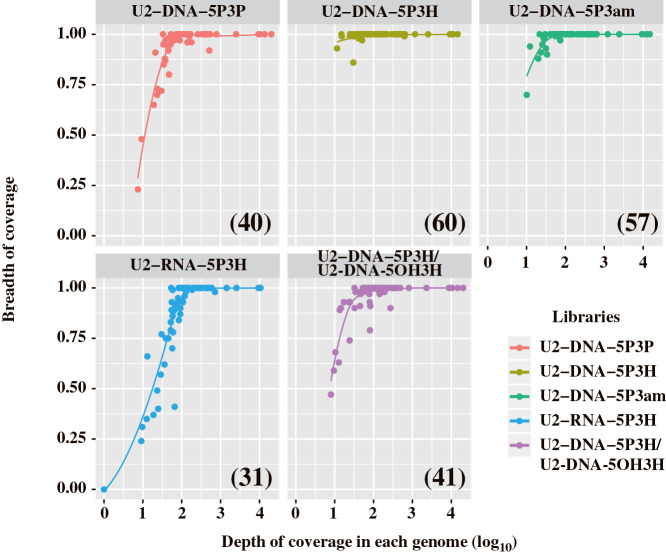
Relationship between the depth and breadth of coverage in FLDS libraries constructed with 5 adaptor types. In the mapping analysis, 67 reference genomes and genome segments of the RNA virome were used as reference sequences. A lower depth of coverage means a smaller number of mapped reads. cDNAs were synthesized with 25 cycles of cDNA amplification. Numbers in parentheses indicate the number of complete reference sequences retrieved by read mapping.

High-quality sequencing libraries may retrieve sequence reads uniformly throughout sequence regions. Therefore, we assessed the quality of libraries by mapping reads on complete RNA viral genomes and genome segments identified from the tested RNA viral consortium (Fig. 2). The read mapping analysis indicated that sequence libraries constructed with U2-DNA-5P3H successfully retrieved most of the reference genome segments in the RNA virome, while other sequence libraries, except for the U2-DNA-5P3am library, did not recover the genome segments at a lower abundance (less than ×100 depth of coverage). Therefore, we concluded that the U2-DNA-5P3H oligonucleotide adaptor needs to be utilized in future studies instead of U2-DNA-5P3P, which was employed in our previous studies.

### Optimization of cDNA amplification and purification

**Fig. 3.**
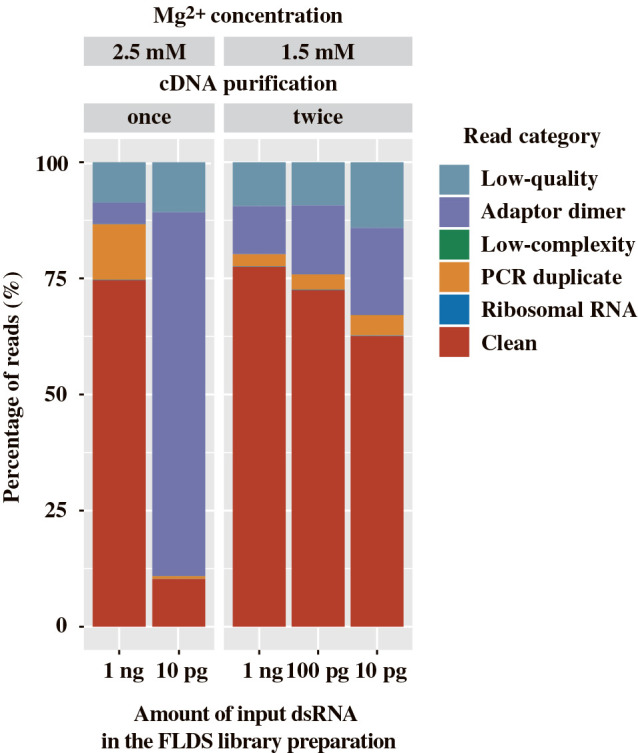
Proportion of reads in sequence libraries constructed from 10 pg to 1‍ ‍ng dsRNA with the U2-DNA-5P3H adaptor. Libraries were generated under either conventional (PCR amplification with 2.5‍ ‍mM Mg^2+^ and cDNA purification performed once) or revised (PCR amplification with 1.5‍ ‍mM Mg^2+^ and cDNA purification performed twice) conditions. All libraries were prepared with 25 cycles of cDNA amplification, except for the library generated from 10 pg dsRNA under conventional conditions (35 cycles).

In addition to the ligation process in FLDS, cDNA amplification also affected sequence quality. Notably, the abundance of high-quality reads (depicted as Clean in Fig. 3) in the U2-DNA-5P3H adaptor library was only 9.7% when constructed from 10 pg of dsRNA. To increase the utility of FLDS with lower amounts of dsRNA, we examined the impact of Mg^2+^ concentrations in the PCR reaction mixture during cDNA amplification and the subsequent purification of amplified cDNA. Reductions in Mg^2+^ concentrations are expected to enhance the specificity of primer annealing. In addition, repeated purification using AMPure XP after cDNA amplification has the potential to exclude short sequences, including the self-ligated products of oligonucleotide adaptors. Therefore, we examined the effects of reductions in the concentration of Mg^2+^ from 2.5 to 1.5‍ ‍mM and repeated cDNA purification twice. FLDS libraries were constructed from 10 pg, 100 pg, and 1‍ ‍ng of mechanically-sheared dsRNA using the U2-DNA-5P3H adaptor. Libraries from subnanogram dsRNA yielded high-quality reads (depicted as Clean in Fig. 3) in 62.6 to 72.4% of all reads, thereby enabling the efficient analysis of the RNA virome. The libraries constructed from subnanogram dsRNA harbored a higher abundance of potential contaminant reads (prokaryotic and eukaryotic reads) (0.57–0.8%) than those constructed from 1‍ ‍ng dsRNA (Table 2). The impact of laboratory contaminants is generally larger in library construction from a very small amount of input dsRNA than that constructed from 1‍ ‍ng dsRNA.

**Fig. 4.**
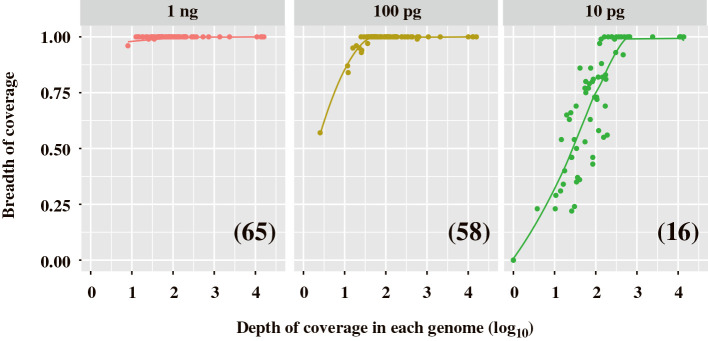
Relationship between the depth and breadth of coverage in FLDS libraries constructed from 10 pg to 1‍ ‍ng dsRNA. In the mapping analysis, 67 reference genomes and genome segments of the RNA virome were used as reference sequences. A lower depth of coverage means a smaller number of mapped reads. cDNAs were synthesized using the U2-DNA-5P3H adaptor with 25 cycles of cDNA amplification. Numbers in parentheses indicate the number of complete reference sequences retrieved by read mapping.

In the assemblies for each library, the number of contigs and total contig lengths were reduced according to the amounts of the dsRNA template: 741‍ ‍kb for 1‍ ‍ng, 662‍ ‍kb for 100 pg, and 270‍ ‍kb for 10 pg (Table 2). Read mapping indicated that sequence reads from the 1-ng dsRNA library retrieved most of the reference genomes and genome segments, whereas retrieval with libraries from lower amounts of dsRNA became more incomplete as template abundance declined (Fig. 4). For example, an complete reference sequence was retrieved in the library constructed from 1‍ ‍ng dsRNA with a depth of coverage of only approximately (approx.) 10, while a depth of coverage of approx. 100 was insufficient to retrieve the same complete sequence in the library from 10 pg dsRNA. This result suggested that the library constructed from 1‍ ‍ng dsRNA had a more uniform depth of coverage than that from 10 pg dsRNA.

Using optimizations of the chemical modifications in oligonucleotide adaptors for cDNA synthesis, PCR conditions for cDNA amplification, and the subsequent purification of amplified cDNA prior to library construction, we successfully constructed sequence libraries from 10 pg of dsRNA. However, their detection sensitivity for low-abundance viruses was inferior to that constructed from 1‍ ‍ng of dsRNA. The optimized conditions of FLDS ver. 3 will enable ranges of applications of this technique for any research related to RNA viromes. However, it is important to note that the diversity of RNA viromes may be affected by the initial input of dsRNA quantity and the number of PCR cycles used for cDNA amplification in FLDS. Therefore, careful interpretations are required when comparing multiple viromes.

## Citation

Hirai, M., Takaki, Y., Kondo, F., Horie, M., Urayama, S., and Nunoura, T. (2021) RNA Viral Metagenome Analysis of Subnanogram dsRNA Using Fragmented and Primer Ligated dsRNA Sequencing (FLDS). *Microbes Environ ***36**: ME20152.

https://doi.org/10.1264/jsme2.ME20152

## Supplementary Material

Supplementary Material

## Figures and Tables

**Table 1.** Assembly statistics for cDNA libraries constructed with 5 types of oligonucleotide adaptors.

	U2-DNA-5P3P	U2-DNA-5P3H	U2-DNA-5P3am	U2-RNA-5P3H	U2-DNA-5P3H/ 5OH3H
cDNA amplification	25 cycles				
Sampled reads	0.8 M	0.8 M	0.8 M	0.8 M	0.8 M
Prokaryotic and eukaryotic reads (%)	0.93	0.76	0.89	1.14	0.89
Assembly					
Number of contigs	366	459	445	295	343
Total size (kb)	479.8	649.7	610.8	382.7	474.7
Average length (bp)	1,310	1,415	1,372	1,297	1,383
N50 (bp)	1,587	1,858	1,637	1,588	1,725
GC content (%)	48.3	48.5	48.8	49.3	48.5
cDNA amplification	35 cycles				
Sampled reads	0.8 M	0.8 M	0.8 M	0.8 M	
Prokaryotic and eukaryotic reads (%)	0.5	0.4	0.53	0.72	
Assembly					
Number of contigs	376	422	420	337	
Total size (kb)	487.7	583	564.5	402	
Average length (bp)	1,296	1,381	1,344	1,193	
N50 (bp)	1,573	1,656	1,542	1,293	
GC content (%)	48.3	48.5	48.4	48.8	

**Table 2.** Assembly statistics for cDNA libraries constructed from 10 pg to 1‍ ‍ng dsRNA and 25 cycles of cDNA amplification.

	1‍ ‍ng	100 pg	10 pg
Sampled reads	0.8 M	0.8 M	0.8 M
Prokaryotic and eukaryotic reads (%)	0.22	0.57	0.80
Assembly			
Number of contigs	474	456	235
Total size (kb)	741.2	661.7	269.9
Average length (bp)	1,563	1,451	1,148
N50 (bp)	2,019	1,756	1,348
GC content (%)	48.2	48.8	49.2
