# Supplementary material for: RNA Viral Metagenome Analysis of Subnanogram dsRNA Using Fragmented and Primer Ligated dsRNA Sequencing (FLDS)
Source: Microbes Environ. 2021 May 1;36(2):ME20152. doi: 10.1264/jsme2.ME20152 (PMC8209451; doi:10.1264/jsme2.ME20152)
Supplement: Supplementary file 1 — Supplementary Material [file 36_20152_s1.pdf]

Supplementary Data

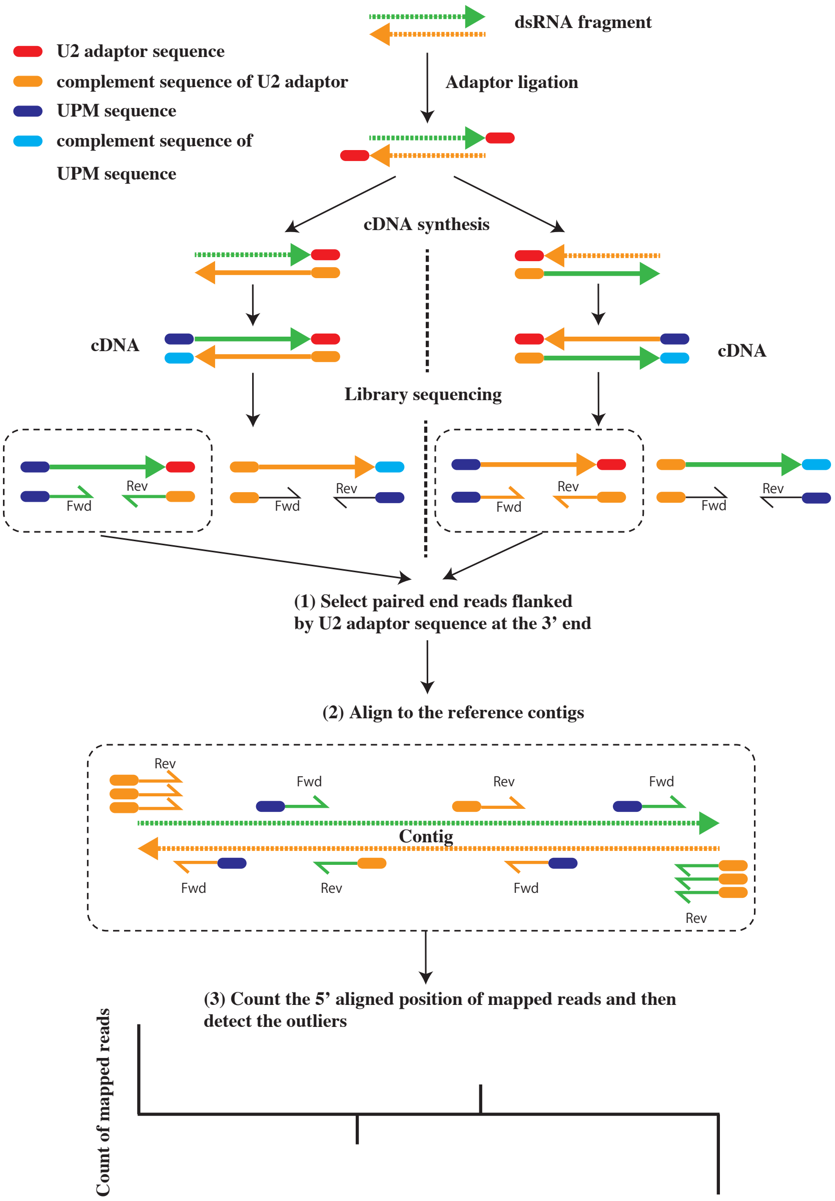

Fig. S1. Workflow for identification of terminal ends of viral genomes and genome segments.

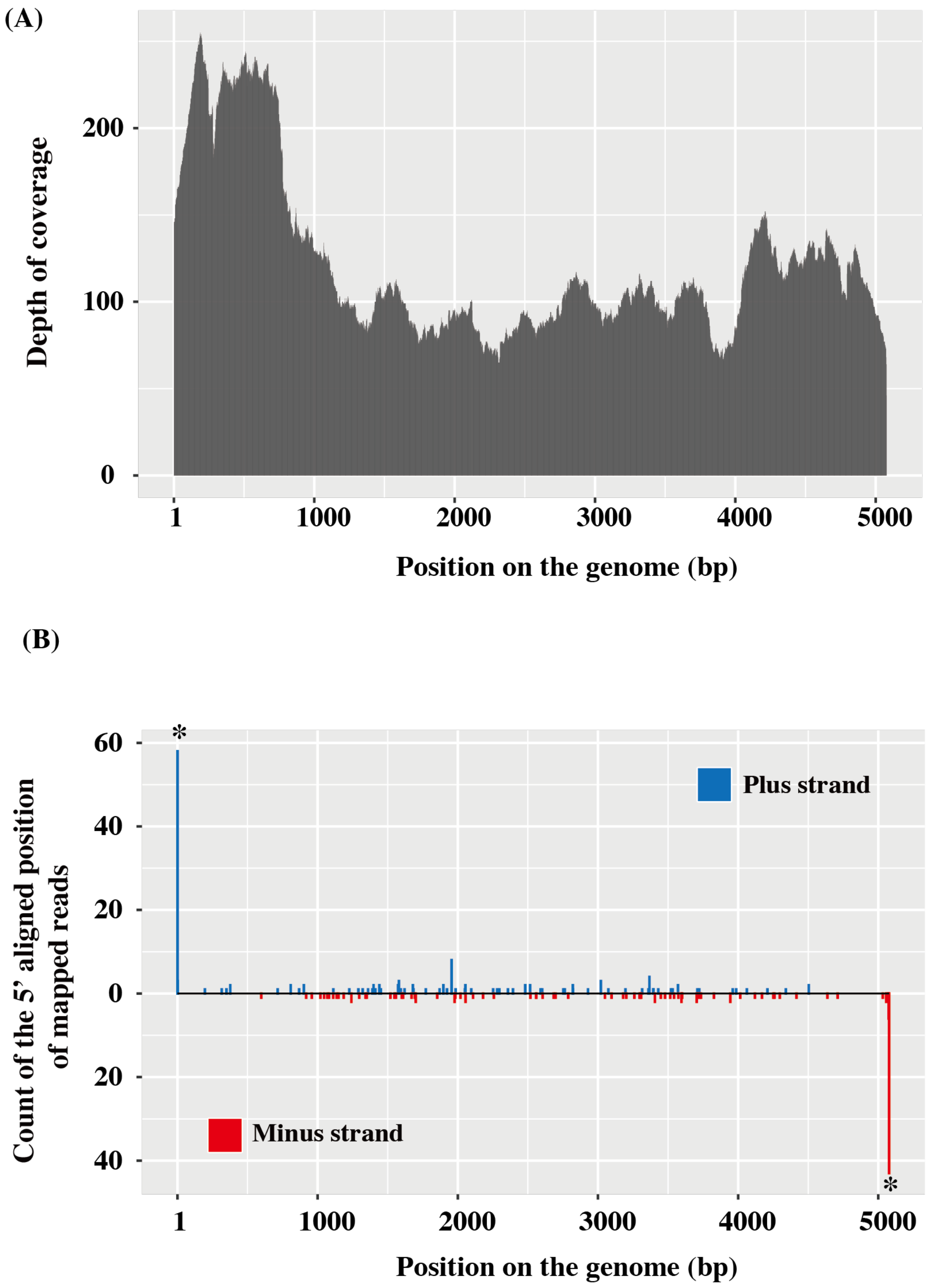

Fig. S2. Sequencing coverage of the paired-end read from a FLDS library on an RNA viral genome (A) and identification of the terminal ends of the genome sequence (B). The asterisk indicates a statistically significant difference ($P < 0.05$) in the Smirnov-Grubbs test.

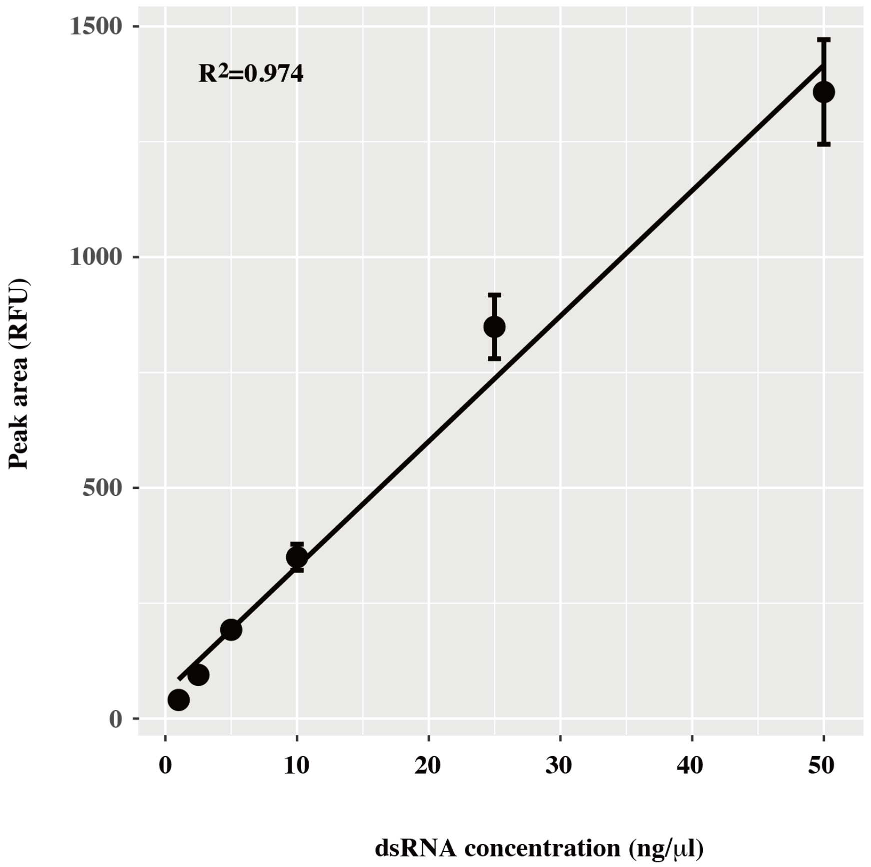

Fig. S3. Standard curve for dsRNA concentration measurements obtained using a Fragment Analyzer. The regression line is a solid black line. RFU: relative fluorescence units.

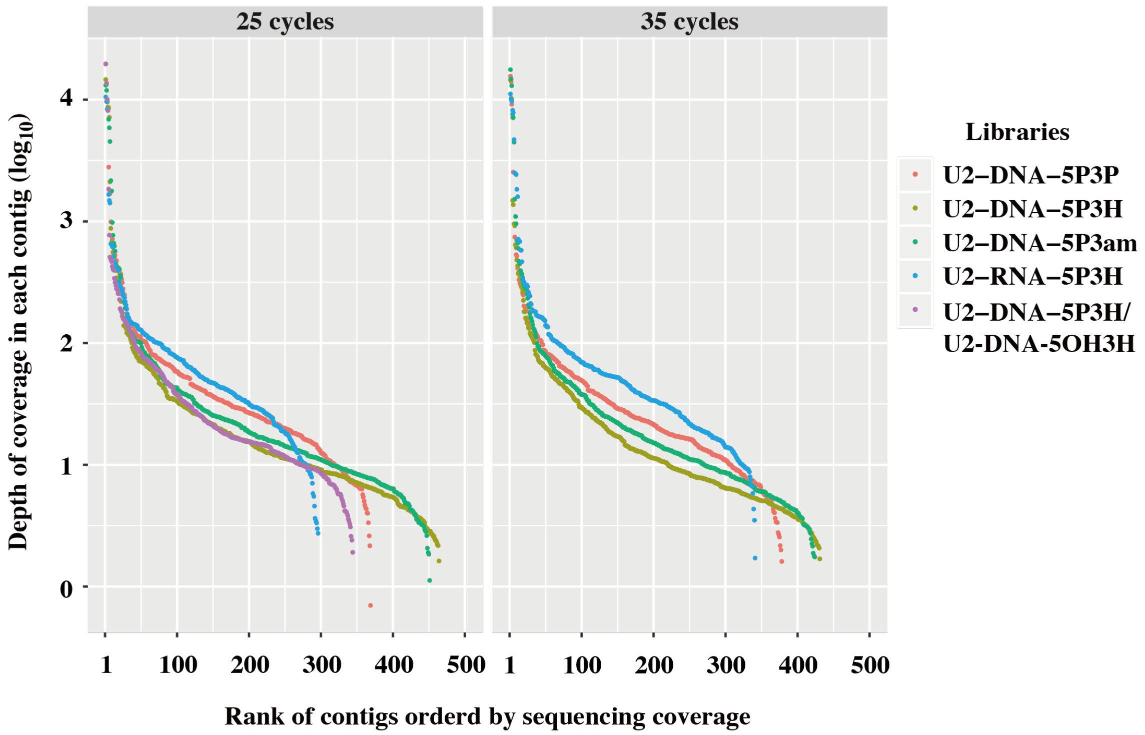

Fig. S4. Rank abundance curves based on sequencing coverage of contigs for the assemblies from the cDNA libraries constructed with 5 adaptors types. Assembly of each library was preformed after removing the prokaryotic and eukaryotic reads from the normalized data set.

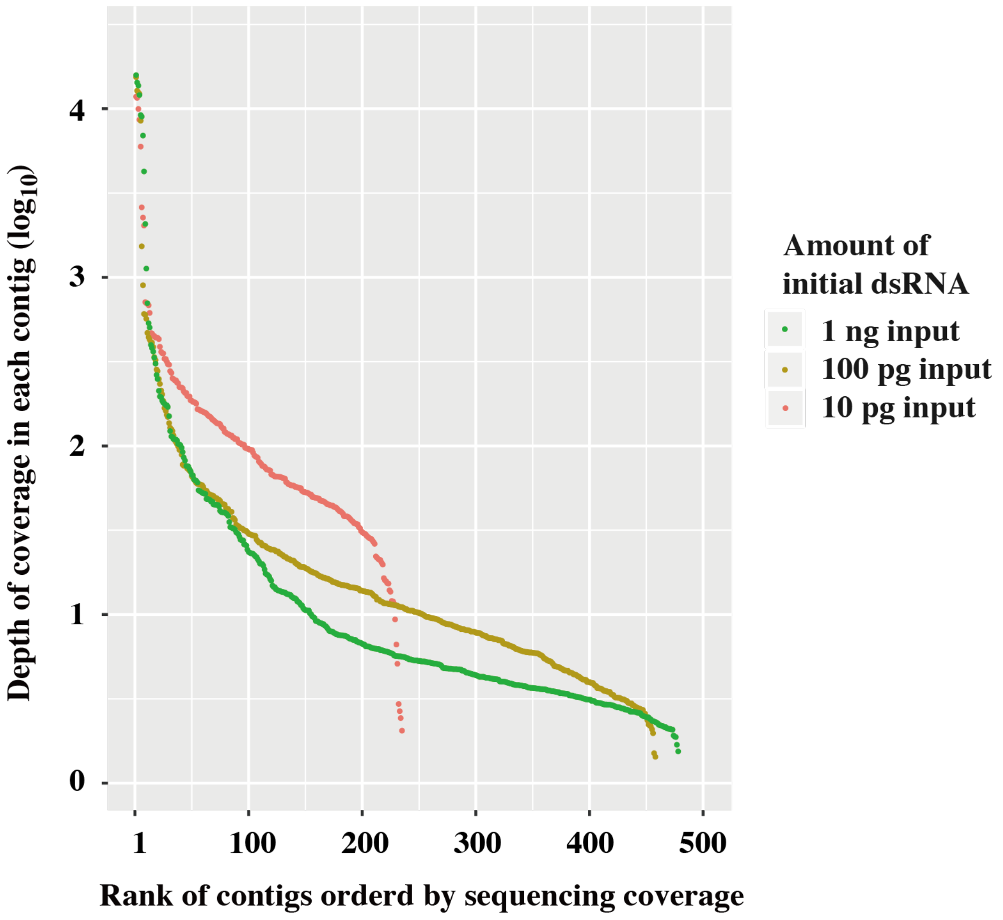

Fig. S5. Rank abundance curves from sequencing coverage of contigs for the assemblies with a various amounts of input dsRNA. Assembly of each library was preformed after removing the prokaryotic and eukaryotic reads from the normalized data set.

Table S1 Putative complete genomes of RNA viruses identified from a diatom.

Accession no.	Length (bp)	Depth of coverage	BLASTX					Taxonomic family	References
			accession	gene	E-value	identity	organism		
AP014891	1562	14408	YP_009333349.1	RdRp	5E-161	48	Beihai picobirna-like virus 8	unclassified RNA viruses	Urayama et al.
AP014890	1734	10753	YP_009333348.1	hypothetical protein	2E-14	25	Beihai picobirna-like virus 7	unclassified RNA viruses	Urayama et al.
AP014892	4026	10448	QKL20127.1	RdRp	7E-29	30	Conidiobolus non-segmented RNA virus 1	unclassified dsRNA viruses	Urayama et al.
AP014914	4568	8624	QKL20127.1	RdRp	4E-19	26	Conidiobolus non-segmented RNA virus 1	unclassified dsRNA viruses	Urayama et al.
AP014915	4786	2465	QKL20127.1	RdRp	2E-15	25	Conidiobolus non-segmented RNA virus 1	unclassified dsRNA viruses	Urayama et al.
BNHM01000033	1572	1010	no hit						This study
AP014916	3458	741	YP_007761588.1	putative coat protein	4E-40	29	Ustilaginoidea virens RNA virus 1	Totiviridae	Urayama et al.
AP014894	4982	614	QLC27590.1	RdRp	4E-70	35	Erysiphe necator associated totivirus 1	Totiviridae	Urayama et al.
AP014910	5907	601	YP_009333150.1	hypothetical protein 2	8E-107	34	Beihai barnacle virus 15	unclassified RNA viruses	Urayama et al.
AP014896	5252	437	YP_009508249.1	RdRp	4E-74	32	Aspergillus foetidus slow virus	Totiviridae	Urayama et al.
AP014920	1986	424	BCD56390.1	RdRp	3E-66	33	Lichen partiti-like RNA virus	Partitiviridae	Urayama et al.
BNHM01000034	2040	415	no hit						This study
AP014909	6635	384	ANR02704.1	RdRp	4E-13	29	Rhizoctonia solani mycovirus	unclassified viruses	Urayama et al.
BNHM01000010	4909	346	VFI65724.1	RdRp	7E-70	30	Umbelopsis ramanniana virus	Totiviridae	This study
BNHM01000031	1863	334	no hit						This study
AP014893	4911	320	VFI65724.1	RdRp	1E-70	31	Umbelopsis ramanniana virus	Totiviridae	Urayama et al.
BNHM01000035	1092	282	no hit						This study
AP014911	5909	233	YP_009333150.1	hypothetical protein 2	5E-101	34	Beihai barnacle virus 15	unclassified RNA viruses	Urayama et al.
AP014897	4939	209	BBZ90082.1	RdRp	3E-73	31	Red algae totivirus 1	Totiviridae	Urayama et al.
AP014917	3190	182	BBZ90080.1	RdRp	0	46	Diatom totivirus 1	Totiviridae	Urayama et al.
AP014919	3325	165	AZT88631.1	RdRp	2E-114	32	Colletotrichum caudatum totivirus 1	Totiviridae	Urayama et al.
AP014900	4844	155	AZT88635.1	RdRp	1E-67	34	Colletotrichum navitas	Totiviridae	Urayama et al.
BNHM01000013	4370	148	no hit						This study
BNHM01000008	5251	141	BBZ90080.1	RdRp	1E-73	35	Diatom totivirus 1	Totiviridae	This study
AP014918	3262	133	YP_009143306.1	putative coat protein	3E-47	28	Magnaporthe oryzae virus 3	Totiviridae	Urayama et al.
AP014899	4660	133	YP_009182167.1	RdRp	7E-67	30	Ustilaginoidea virens RNA virus 5	Totiviridae	Urayama et al.
AP014903	5160	128	BBZ90080.1	RdRp	2E-134	35	Diatom totivirus 1	Totiviridae	Urayama et al.
AP014913	4586	120	AEP83726.1	RdRp	2E-19	31	Tuber excavatum mitovirus	Mitoviridae	Urayama et al.
BNHM01000021	3463	114	QKL20127.1	RdRp	1E-24	31	Conidiobolus non-segmented RNA virus 1	unclassified dsRNA viruses	This study
BNHM01000016	3392	106	no hit						This study
AP014898	5327	100	NP_624332.2	RdRp	8E-120	36	Gremmeniella abietina RNA virus L1	Totiviridae	Urayama et al.
BNHM01000028	2317	100	no hit						This study
BNHM01000018	3468	89	no hit						This study
AP014902	5082	86	BBZ90080.1	RdRp	6E-140	35	Diatom totivirus 1	Totiviridae	Urayama et al.
AP014905	4671	85	VFI65724.1	RdRp	4E-66	33	Umbelopsis ramanniana virus	Totiviridae	Urayama et al.
AP014907	1490	83	ASM94044.1	putative capsid	3E-110	49	Barns Ness breadcrumb sponge partiti-like virus 1	Partitiviridae	Urayama et al.
BNHM01000007	5326	82	NP_624332.2	RdRp	7E-119	37	Gremmeniella abietina RNA virus L1	Totiviridae	This study
BNHM01000029	1956	81	no hit						This study
AP014906	1576	79	QBC66121.1	RdRp	2E-98	41	Cucumis melo cryptic virus	Partitiviridae	Urayama et al.
AP014904	5941	78	VFI65724.1	RdRp	3E-45	26	Umbelopsis ramanniana virus	Totiviridae	Urayama et al.
AP014908	12940	77	AZO92732.1	polyprotein	2E-140	24	Arthrocladiella mougeotii alphaendornavirus	Endornaviridae	Urayama et al.
BNHM01000015	3565	76	NP_047557.1	coat protein	7E-32	26	Sphaeropsis sapinea RNA	Totiviridae	This study
AP014895	4979	74	YP_007761589.1	RdRp	5E-73	34	Ustilaginoidea virens RNA virus 1	Totiviridae	Urayama et al.
AP014901	4845	74	AZT88635.1	RdRp	1E-69	34	Colletotrichum navitas	Totiviridae	Urayama et al.
BNHM01000001	12951	73	AZO92732.1	polyprotein	7E-141	26	Arthrocladiella mougeotii alphaendornavirus	Endornaviridae	This study
AP014912	11413	71	QIP68012.1	RdRp	2E-84	30	Bremia lactucae associated ssRNA virus 1	unclassified ssRNA viruses	Urayama et al.
BNHM01000005	5079	70	BBZ90080.1	RdRp	6E-144	37	Diatom totivirus 1	Totiviridae	This study
BNHM01000017	3512	65	YP_009032632.1	coat protein	2E-15	31	Beauveria bassiana victorivirus NZL/1980	Totiviridae	This study
BNHM01000019	3169	60	AZT88637.1	RdRp	1E-124	33	Colletotrichum zoysiae	Totiviridae	This study
BNHM01000004	5942	60	VFI65724.1	RdRp	4E-43	26	Umbelopsis ramanniana virus	Totiviridae	This study
BNHM01000011	4659	58	VFI65724.1	RdRp	2E-67	33	Umbelopsis ramanniana virus	Totiviridae	This study
BNHM01000020	4824	55	VFI65724.1	RdRp	6E-66	31	Umbelopsis ramanniana virus	Totiviridae	This study
BNHM01000003	6103	48	APG78191.1	RdRp	1E-17	27	Beihai picobirna-like virus 10	unclassified RNA viruses	This study
BNHM01000002	7161	46	AJE29745.1	RdRp	5E-66	31	Rhizoctonia fumigata	unclassified viruses	This study
BNHM01000012	5910	44	YP_009333150.1	hypothetical protein 2	2E-98	32	Beihai barnacle virus 15	unclassified RNA viruses	This study
BNHM01000024	3071	41	no hit						This study
BNHM01000014	6135	41	QGA70930.1	RdRp	4E-106	32	Ahus virus	Totiviridae	This study
BNHM01000009	4907	38	YP_009272905.1	RdRp	1E-74	34	Fusarium poae victorivirus 1	Totiviridae	This study
BNHM01000006	14823	36	QIP68012.1	RdRp	6E-143	28	Bremia lactucae associated ssRNA virus 1	unclassified ssRNA viruses	This study
BNHM01000023	3062	35	no hit						This study
BNHM01000026	2885	33	no hit						This study
BNHM01000032	1839	31	no hit						This study
BNHM01000030	5084	21	BBZ90080.1	RdRp	6E-141	37	Diatom totivirus 1	Totiviridae	This study
BNHM01000025	2884	21	no hit						This study
BNHM01000022	3119	20	QIJ70073.1	RdRp	3E-121	31	Swanson narna-like virus	Narnaviridae	This study
BNHM01000027	2373	17	no hit						This study
BNHM01000036	6284	16	APG76021.1	hypothetical protein	1.0E-07	23	Hubei toti-like virus 11	unclassified RNA viruses	This study

Depth of coverage were estimated from the U2-DNA-5P3H library. Abbreviation: RdRP, RNA dependent RNA polymerase.
